# Supplementary material for: Identification of Genes Responsible for the Synthesis of Glycitein Isoflavones in Soybean Seeds
Source: Plants (Basel). 2024 Jan 5;13(2):156. doi: 10.3390/plants13020156 (PMC10818676; doi:10.3390/plants13020156)
Supplement: Supplementary file 1 [file plants-13-00156-s001.zip › plants-2736886-supplementary.pptx]

## Slide 1
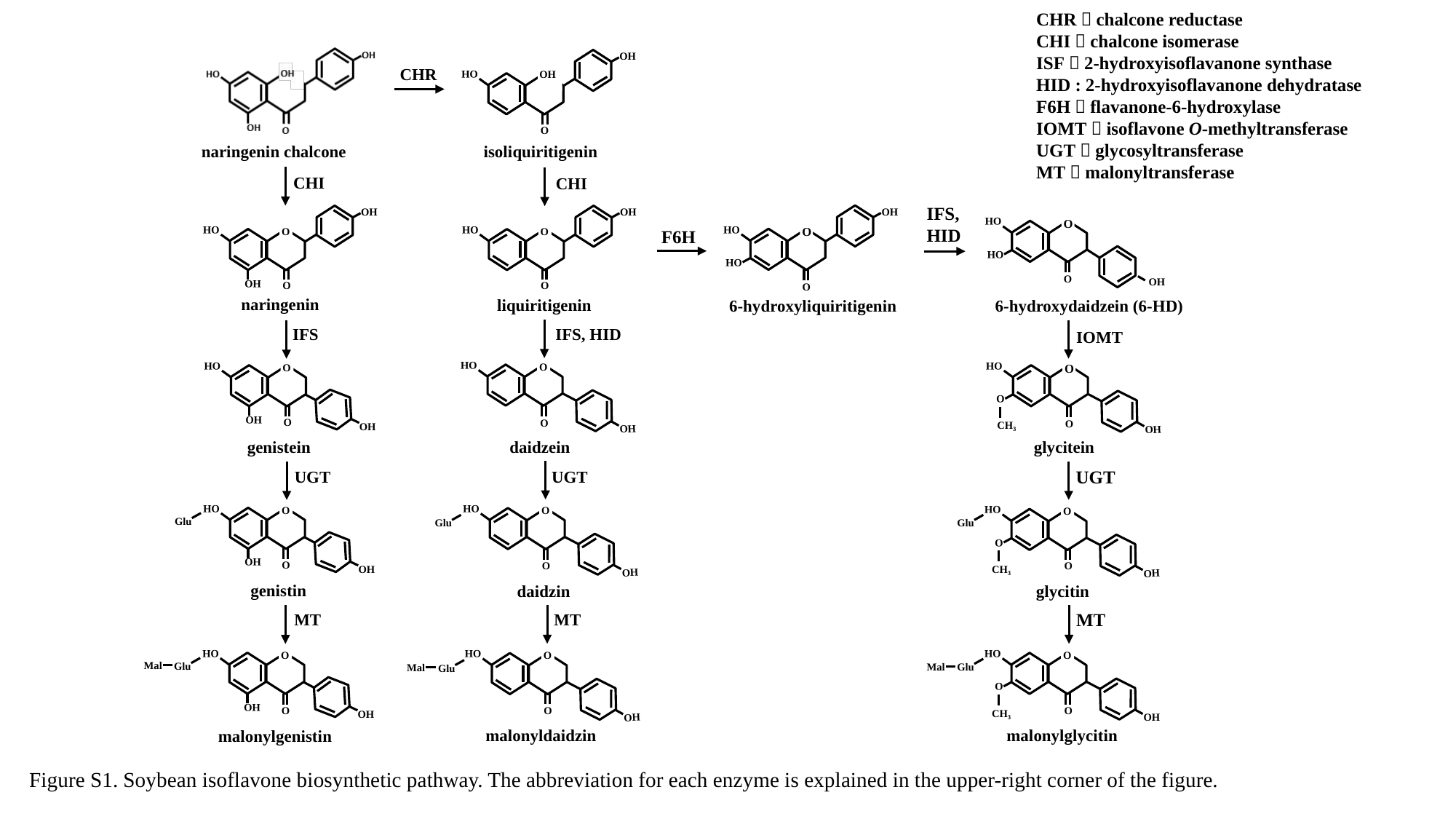

CHR：chalcone reductase
CHI：chalcone isomerase
ISF：2-hydroxyisoflavanone synthase
HID : 2-hydroxyisoflavanone dehydratase
F6H：flavanone-6-hydroxylase
IOMT：isoflavone O-methyltransferase
UGT：glycosyltransferase
MT：malonyltransferase
naringenin chalcone
CHI
OH
OH
HO
O
O
naringenin
IFS
OH
HO
O
OH
O
genistein
UGT
OH
HO
O
OH
Glu
O
genistin
MT
OH
HO
O
OH
Glu
Mal
O
malonylgenistin
OH
OH
OH
O
isoliquiritigenin
CHR
CHI
OH
OH
O
O
liquiritigenin
IFS, HID
OH
O
OH
O
daidzein
UGT
OH
O
OH
Glu
O
daidzin
MT
OH
O
OH
Mal
Glu
O
malonyldaidzin
IFS,
HID
OH
O
OH
O
OH
6-hydroxydaidzein (6-HD)
IOMT
OH
O
O
OH
CH₃
O
glycitein
UGT
OH
O
O
OH
CH₃
Glu
O
glycitin
MT
OH
O
O
OH
CH₃
Glu
Mal
O
malonylglycitin
OH
OH
OH
O
O
6-hydroxyliquiritigenin
F6H
Figure S1. Soybean isoflavone biosynthetic pathway. The abbreviation for each enzyme is explained in the upper-right corner of the figure.

## Slide 2
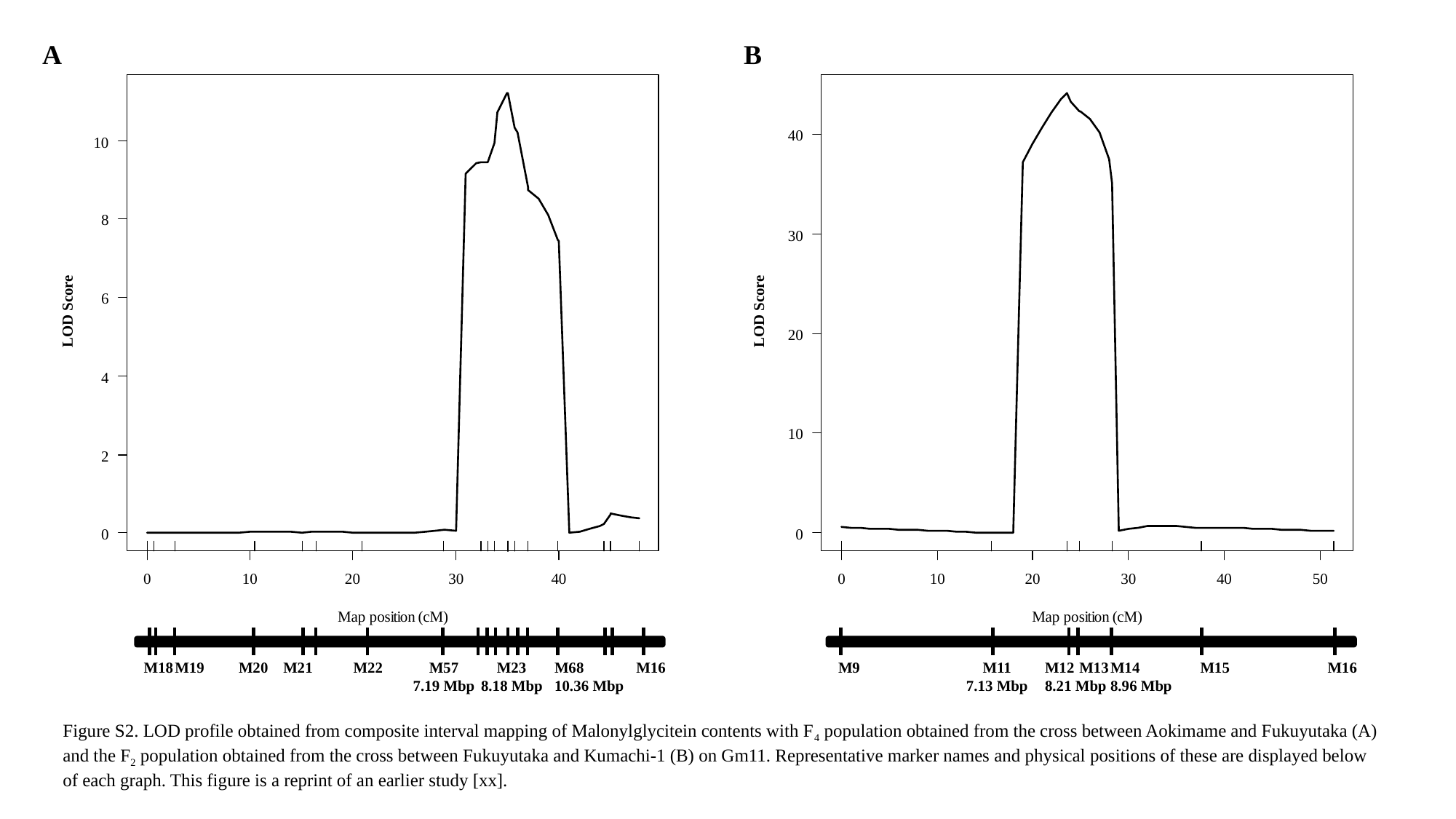

A
B
LOD Score
LOD Score
M18
M19
M20
M21
M22
M57
7.19 Mbp
M23
8.18 Mbp
M68
10.36 Mbp
M16
M9
M11
7.13 Mbp
M12
8.21 Mbp
M13
M14
8.96 Mbp
M15
M16
Figure S2. LOD profile obtained from composite interval mapping of Malonylglycitein contents with F4 population obtained from the cross between Aokimame and Fukuyutaka (A) and the F2 population obtained from the cross between Fukuyutaka and Kumachi-1 (B) on Gm11. Representative marker names and physical positions of these are displayed below of each graph. This figure is a reprint of an earlier study [xx].

## Slide 3
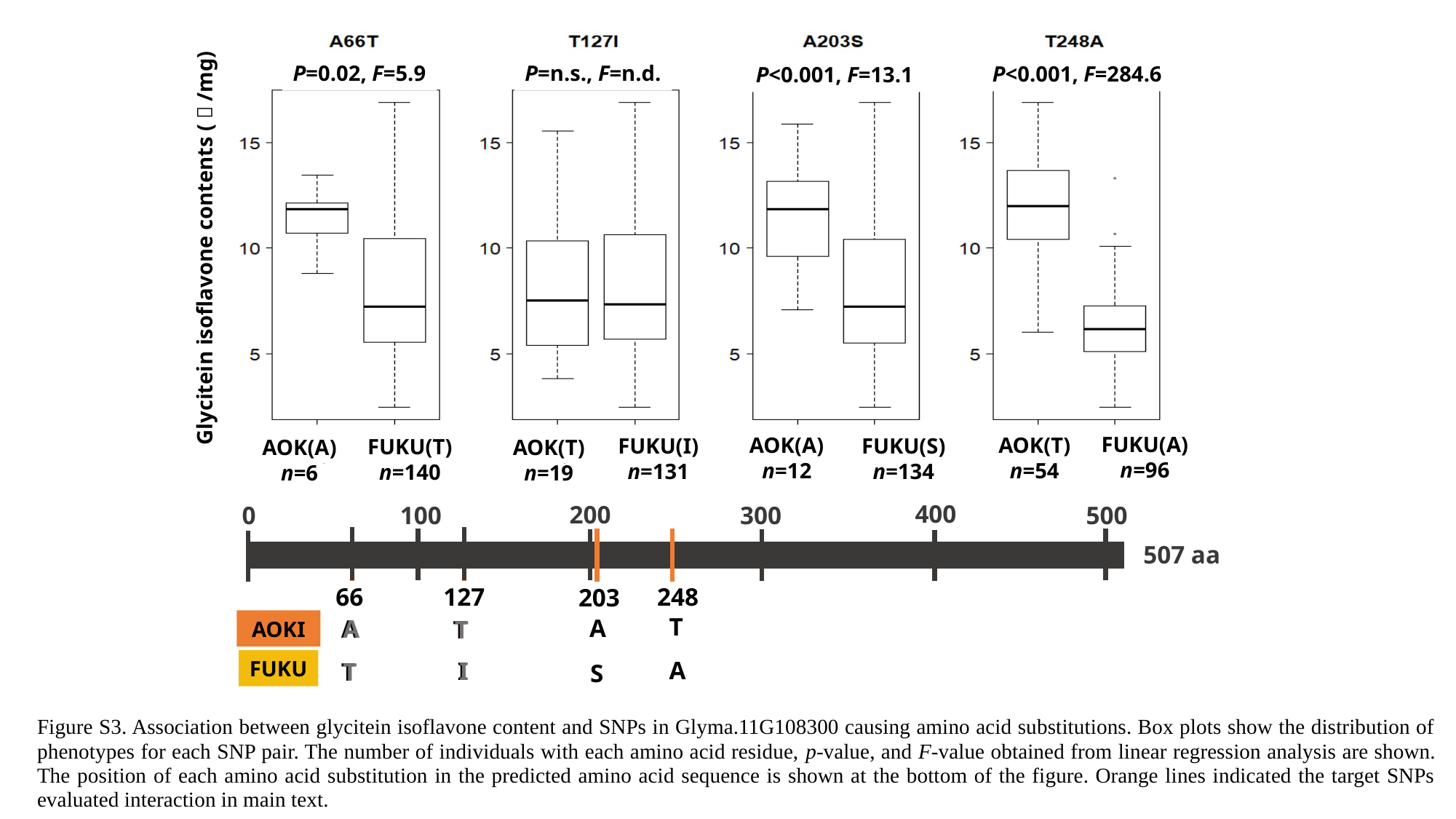

FUKU(A)
n=96
AOK(T)
n=54
AOK(A)
n=12
FUKU(S)
n=134
FUKU(I)
n=131
FUKU(T)
n=140
AOK(A)
n=6
AOK(T)
n=19
P=0.02, F=5.9
P=n.s., F=n.d.
P<0.001, F=284.6
P<0.001, F=13.1
Glycitein isoflavone contents (㎍/mg)
400
200
500
0
100
300
507 aa
66
127
248
203
T
A
A
S
A
T
I
T
A
T
I
T
AOKI
FUKU
Figure S3. Association between glycitein isoflavone content and SNPs in Glyma.11G108300 causing amino acid substitutions. Box plots show the distribution of phenotypes for each SNP pair. The number of individuals with each amino acid residue, p-value, and F-value obtained from linear regression analysis are shown. The position of each amino acid substitution in the predicted amino acid sequence is shown at the bottom of the figure. Orange lines indicated the target SNPs evaluated interaction in main text.

## Slide 4
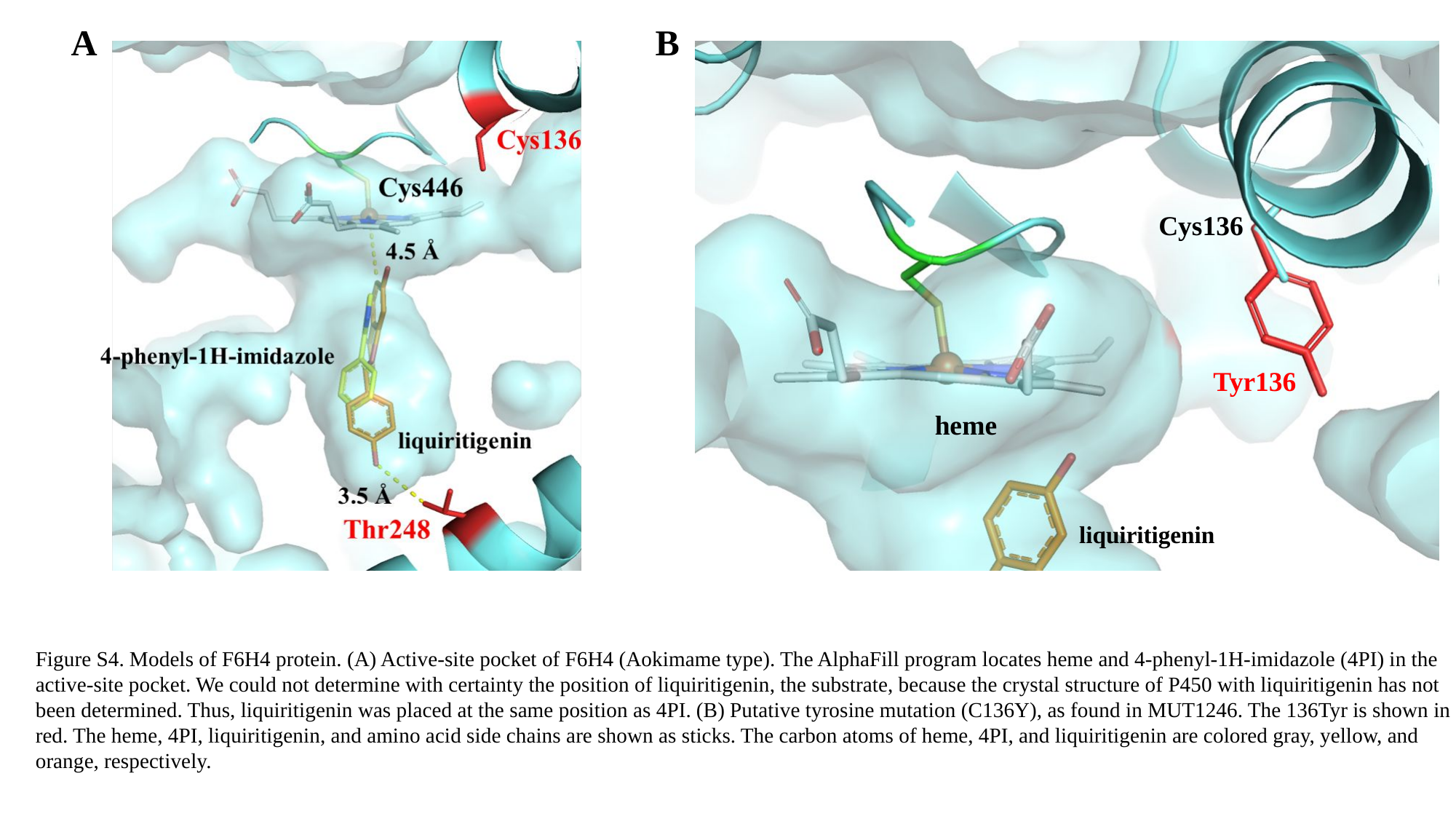

A
B
Cys136
Tyr136
heme
liquiritigenin
Figure S4. Models of F6H4 protein. (A) Active-site pocket of F6H4 (Aokimame type). The AlphaFill program locates heme and 4-phenyl-1H-imidazole (4PI) in the active-site pocket. We could not determine with certainty the position of liquiritigenin, the substrate, because the crystal structure of P450 with liquiritigenin has not been determined. Thus, liquiritigenin was placed at the same position as 4PI. (B) Putative tyrosine mutation (C136Y), as found in MUT1246. The 136Tyr is shown in red. The heme, 4PI, liquiritigenin, and amino acid side chains are shown as sticks. The carbon atoms of heme, 4PI, and liquiritigenin are colored gray, yellow, and orange, respectively.

## Slide 5
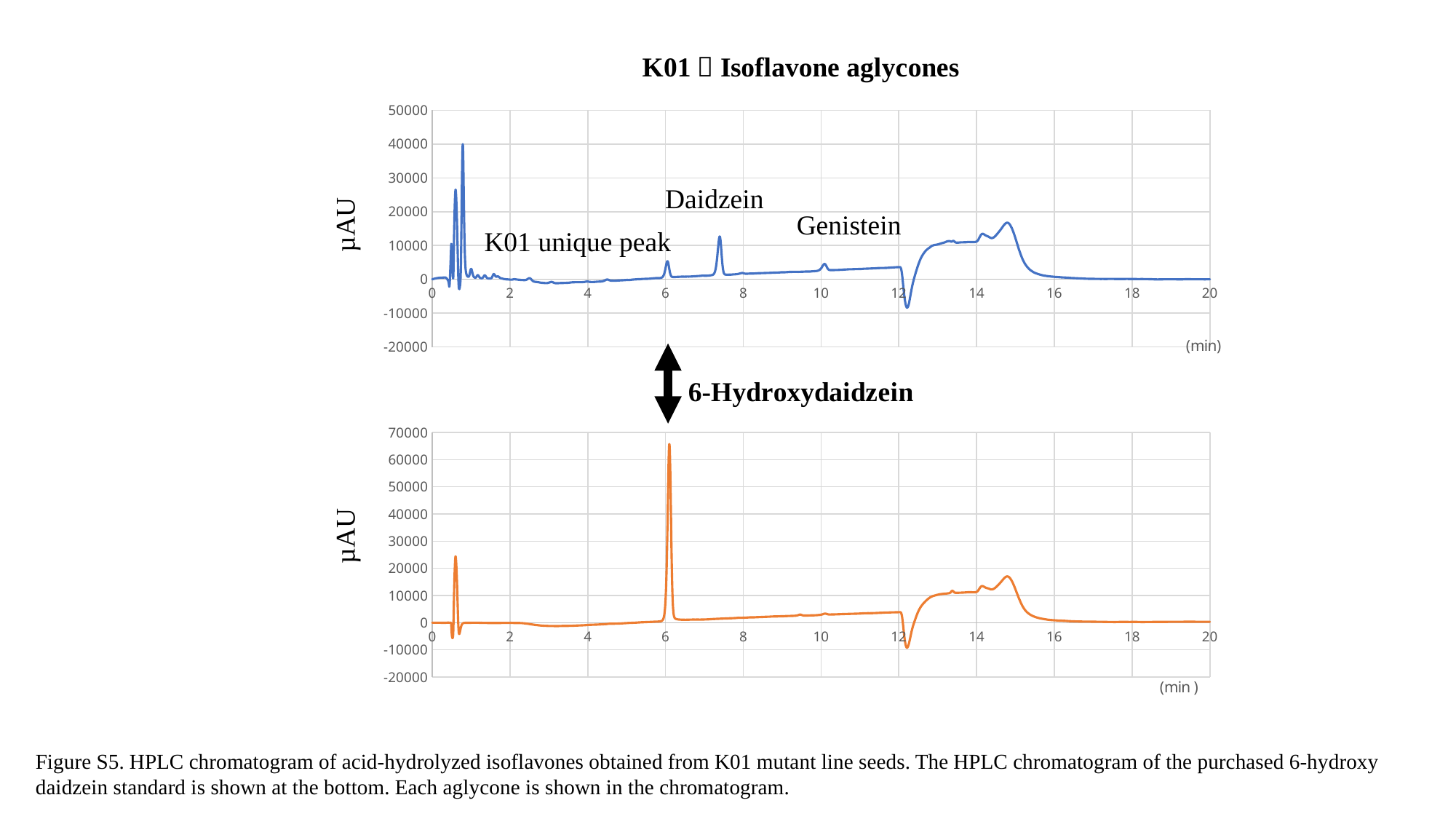

### Chart: K01：Isoflavone aglycones
| Category | Intensity |
|---|---|Daidzein
µAU
Genistein
K01 unique peak
### Chart: 6-Hydroxydaidzein
| Category | Intensity |
|---|---|µAU
Figure S5. HPLC chromatogram of acid-hydrolyzed isoflavones obtained from K01 mutant line seeds. The HPLC chromatogram of the purchased 6-hydroxy daidzein standard is shown at the bottom. Each aglycone is shown in the chromatogram.

## Slide 6
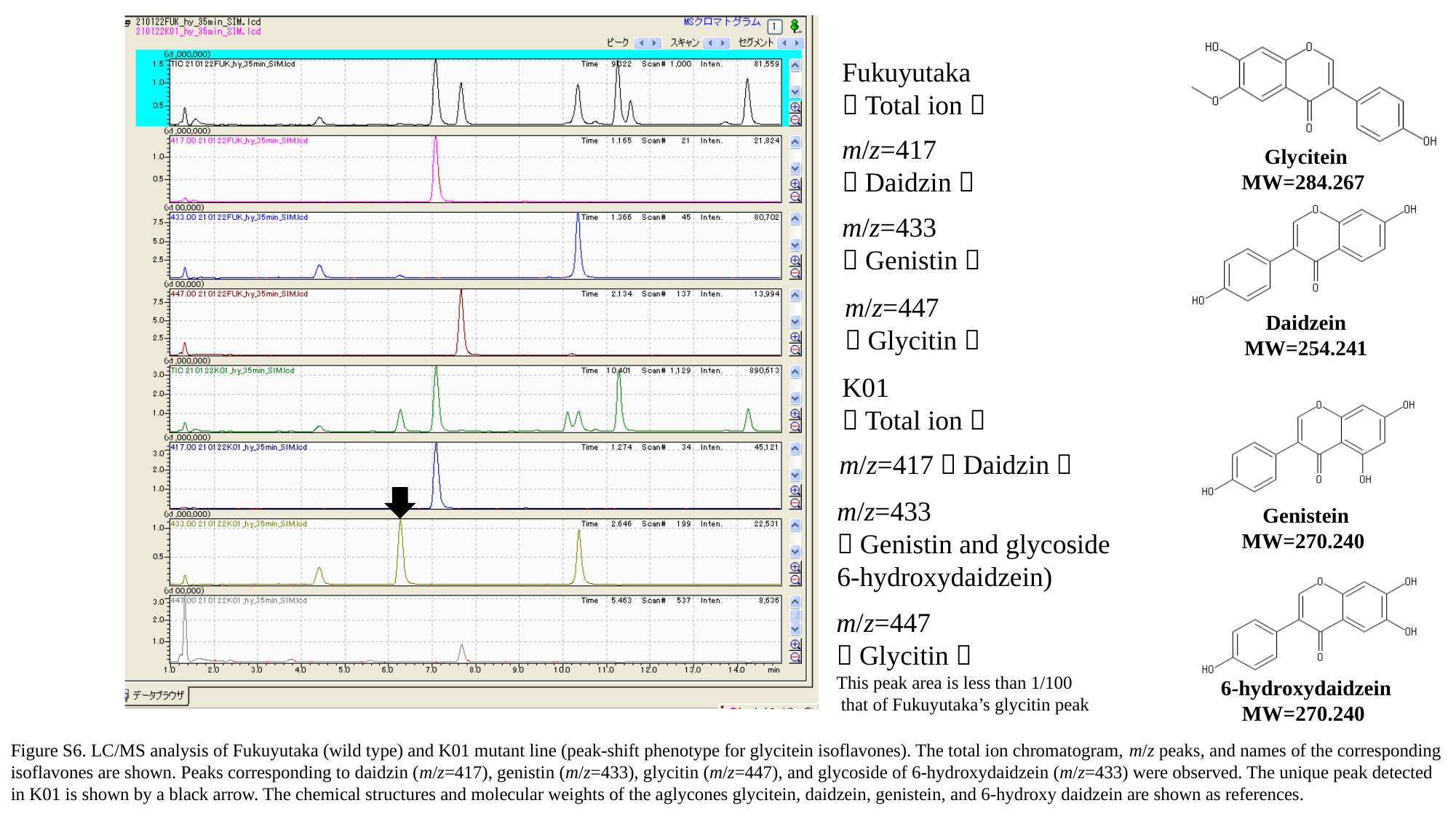

Fukuyutaka
（Total ion）
m/z=417
（Daidzin）
Glycitein
MW=284.267
m/z=433
（Genistin）
m/z=447
（Glycitin）
Daidzein
MW=254.241
K01
（Total ion）
m/z=417（Daidzin）
m/z=433
（Genistin and glycoside
6-hydroxydaidzein)
Genistein
MW=270.240
m/z=447
（Glycitin）
This peak area is less than 1/100
 that of Fukuyutaka’s glycitin peak
6-hydroxydaidzein
MW=270.240
Figure S6. LC/MS analysis of Fukuyutaka (wild type) and K01 mutant line (peak-shift phenotype for glycitein isoflavones). The total ion chromatogram, m/z peaks, and names of the corresponding isoflavones are shown. Peaks corresponding to daidzin (m/z=417), genistin (m/z=433), glycitin (m/z=447), and glycoside of 6-hydroxydaidzein (m/z=433) were observed. The unique peak detected in K01 is shown by a black arrow. The chemical structures and molecular weights of the aglycones glycitein, daidzein, genistein, and 6-hydroxy daidzein are shown as references.

## Slide 7
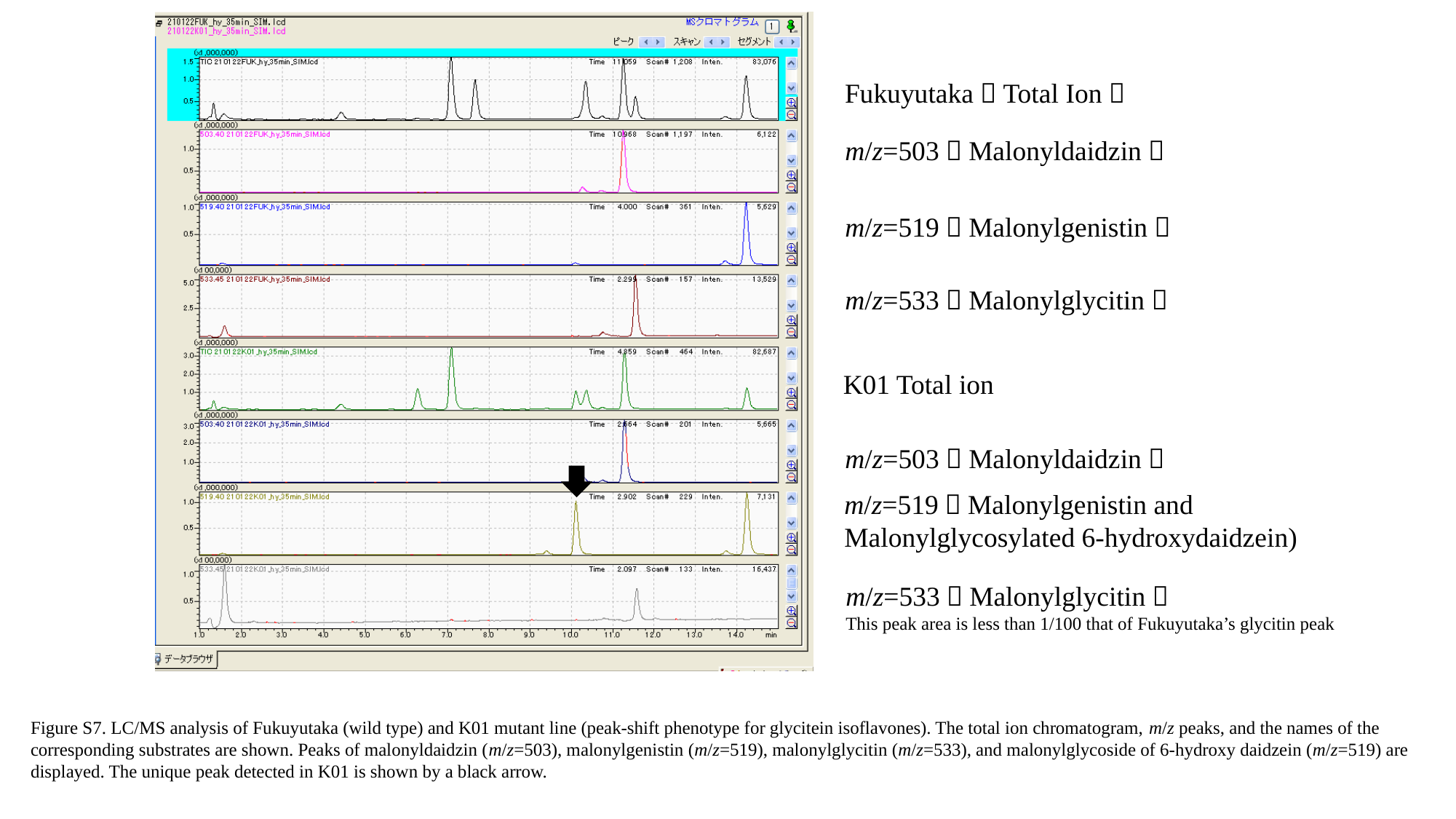

Fukuyutaka（Total Ion）
m/z=503（Malonyldaidzin）
m/z=519（Malonylgenistin）
m/z=533（Malonylglycitin）
K01 Total ion
m/z=503（Malonyldaidzin）
m/z=519（Malonylgenistin and
Malonylglycosylated 6-hydroxydaidzein)
m/z=533（Malonylglycitin）
This peak area is less than 1/100 that of Fukuyutaka’s glycitin peak
Figure S7. LC/MS analysis of Fukuyutaka (wild type) and K01 mutant line (peak-shift phenotype for glycitein isoflavones). The total ion chromatogram, m/z peaks, and the names of the corresponding substrates are shown. Peaks of malonyldaidzin (m/z=503), malonylgenistin (m/z=519), malonylglycitin (m/z=533), and malonylglycoside of 6-hydroxy daidzein (m/z=519) are displayed. The unique peak detected in K01 is shown by a black arrow.

## Slide 8
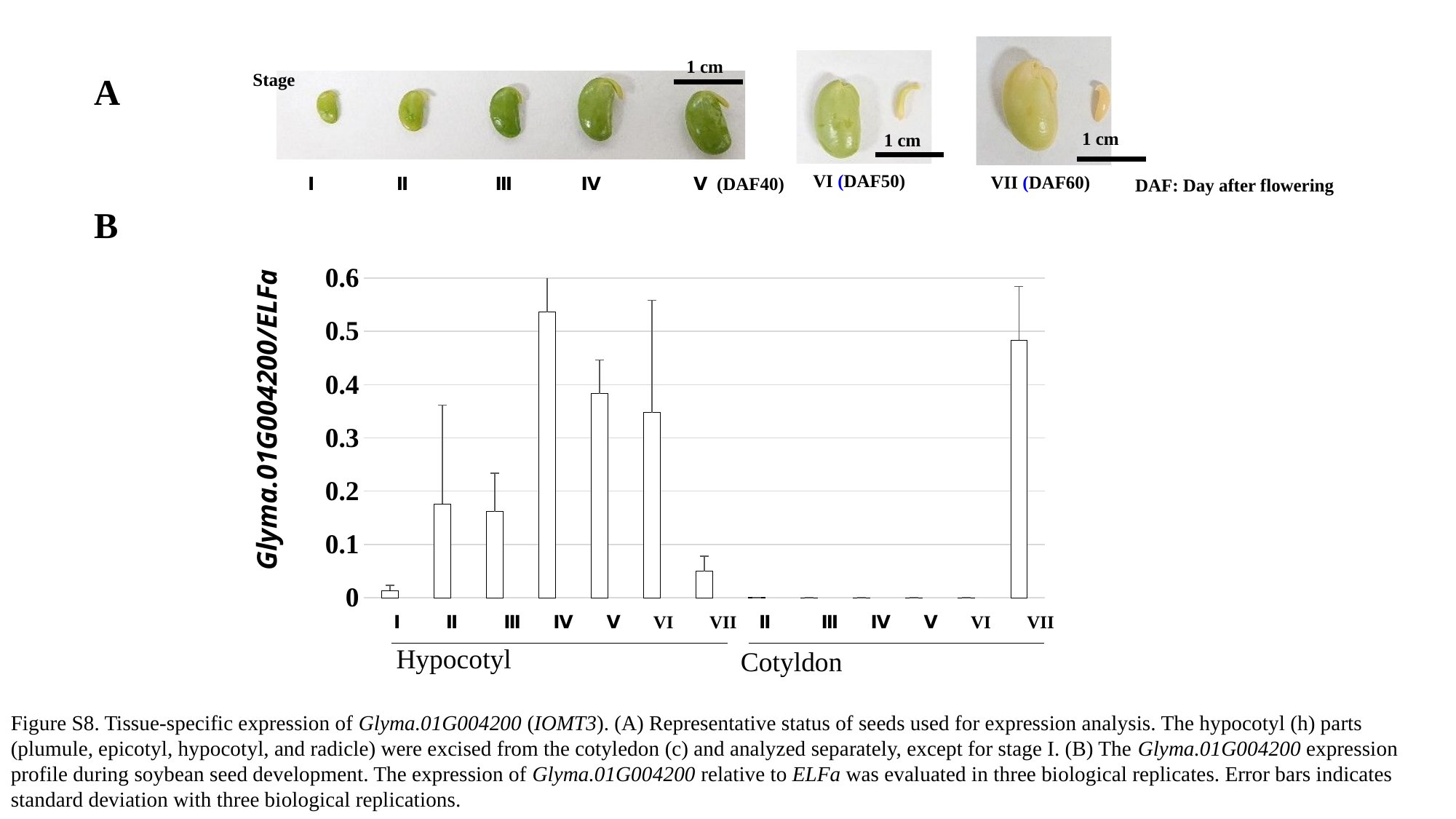

1 cm
Stage
VI (DAF50)
VII (DAF60)
DAF: Day after flowering
1 cm
1 cm
A
Ⅰ 　　　　Ⅱ 　　　　 Ⅲ 　　　Ⅳ 　　　Ⅴ (DAF40)
B
### Chart
| Category | Taro1 |
|---|---|
| 1 | 0.01355096688562374 |
| 2h | 0.17619074865553605 |
| 3h | 0.16199460277792818 |
| 4h | 0.5365189656207274 |
| 5h | 0.38322433577487763 |
| 6h | 0.3477151843286235 |
| 7h | 0.04959526602122582 |
| 2c | 0.000244140625 |
| 3c | 0.0 |
| 4c | 0.0 |
| 5c | 0.0 |
| 6c | 0.0 |
| 7c | 0.48329012233385527 |Glyma.01G004200/ELFa
Ⅰ 　　Ⅱ 　　 Ⅲ 　Ⅳ Ⅴ VI VII
Ⅱ 　　 Ⅲ 　Ⅳ Ⅴ VI VII
Hypocotyl
Cotyldon
Figure S8. Tissue-specific expression of Glyma.01G004200 (IOMT3). (A) Representative status of seeds used for expression analysis. The hypocotyl (h) parts (plumule, epicotyl, hypocotyl, and radicle) were excised from the cotyledon (c) and analyzed separately, except for stage I. (B) The Glyma.01G004200 expression profile during soybean seed development. The expression of Glyma.01G004200 relative to ELFa was evaluated in three biological replicates. Error bars indicates standard deviation with three biological replications.

## Slide 9
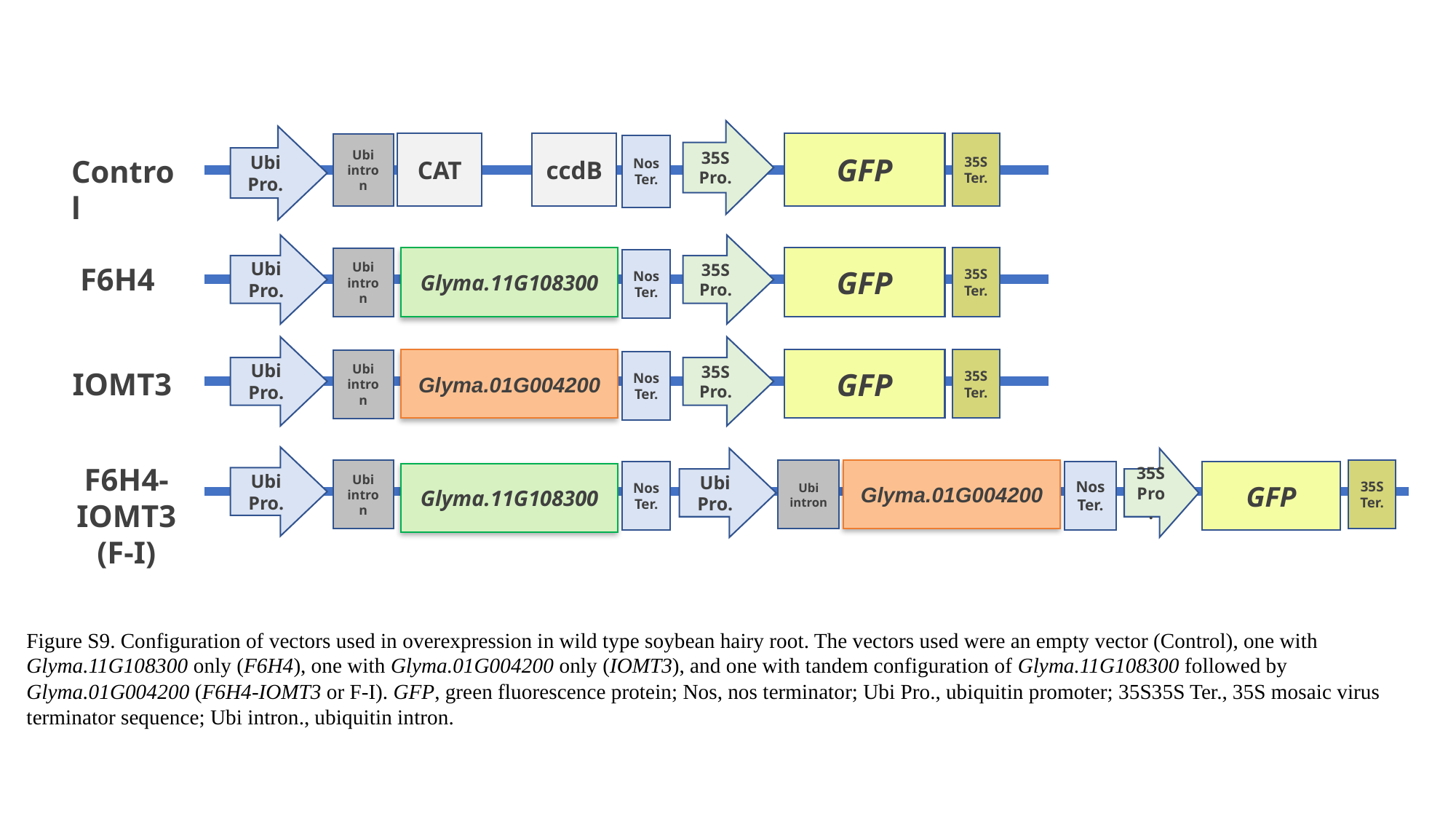

35S Pro.
Ubi
Pro.
CAT
ccdB
GFP
35S
Ter.
Ubi
intron
Nos
Ter.
Control
Ubi
Pro.
35S Pro.
GFP
35S
Ter.
Glyma.11G108300
Ubi
intron
Nos
Ter.
F6H4
Ubi
Pro.
35S Pro.
GFP
35S
Ter.
Glyma.01G004200
Ubi
intron
Nos
Ter.
IOMT3
Ubi
Pro.
35S Pro.
Ubi
Pro.
F6H4-IOMT3
(F-I)
35S
Ter.
Glyma.01G004200
Ubi
intron
Ubi
intron
Nos
Ter.
Nos
Ter.
GFP
Glyma.11G108300
Figure S9. Configuration of vectors used in overexpression in wild type soybean hairy root. The vectors used were an empty vector (Control), one with Glyma.11G108300 only (F6H4), one with Glyma.01G004200 only (IOMT3), and one with tandem configuration of Glyma.11G108300 followed by Glyma.01G004200 (F6H4-IOMT3 or F-I). GFP, green fluorescence protein; Nos, nos terminator; Ubi Pro., ubiquitin promoter; 35S35S Ter., 35S mosaic virus terminator sequence; Ubi intron., ubiquitin intron.
